# Impact of Bariatric Surgery on Post-Transplant Outcomes in Solid Organ Recipients: A Retrospective Cohort Study

**DOI:** 10.3390/jcm15030954

**Published:** 2026-01-24

**Authors:** Leandro Sierra, Kanisha Bahierathan, Maria Ortega Abad, Akash Khurana, Arjun Chatterjee, Roma Patel, Stephen Firkins, Roberto Simons-Linares

**Affiliations:** 1 Department of Medicine, Cleveland Clinic Foundation, Cleveland, OH 44195, USA; 2 Department of Medicine, Case Western Reserve University School of Medicine, Cleveland, OH 44106, USA; ksb128@case.edu; 3 Department of Endocrinology and Metabolism, Cleveland Clinic Foundation, Cleveland, OH 44195, USA; ortegam3@ccf.org; 4 Department of Gastroenterology and Hepatology, Cleveland Clinic Foundation, Cleveland, OH 44195, USA; khurana@ccf.org (A.K.); chattea2@ccf.org (A.C.); patelr43@ccf.org (R.P.); firkins@ccf.org (S.F.); 5 Division of Gastroenterology, Loyola University Medical Center, Maywood, IL 60153, USA

**Keywords:** bariatric surgery, solid organ transplantation, graft rejection, obesity, transplant outcomes, sleeve gastrectomy, kidney transplant, liver transplant

## Abstract

**Background/Objectives:** Obesity affects over 40% of solid organ transplant candidates, increasing graft complications. Bariatric surgery remains underutilized in this population due to safety concerns. We sought to evaluate predictors of graft success among patients with and without a history of bariatric surgery. **Methods:** We utilized the Nationwide Inpatient Sample (2015–2020) to identify adult solid organ transplant recipients with or without a history of bariatric surgery. Propensity score matching (2:1) was performed. The primary outcome was a composite of graft-related complications, including acute or chronic rejection, graft failure, and organ-specific transplant complications. **Results:** Among 196,871 transplant recipients, 2670 (1.4%) had a bariatric surgery history. After matching, 2530 bariatric surgery patients (age 55.6 ± 11.3 years, 37.5% female, 29.0% obese) were compared with 4817 controls (age 56.3 ± 13.9 years, 36.0% female. 29.1% obese). Bariatric surgery patients had significantly lower composite graft complications (7.7% vs. 10.5%; *p* < 0.001), driven by reductions in chronic graft rejection (2.1% vs. 3.1%; *p* = 0.01), kidney complications (6.2% vs. 8.4%; *p* < 0.001), and pancreas complications (0.2% vs. 0.6%; *p* = 0.004). Multivariate analysis showed bariatric surgery was independently associated with 23% reduced odds of graft complications (OR 0.77; 95% CI 0.61–0.96; *p* = 0.02). **Conclusions:** Bariatric surgery was independently associated with reduced graft-related complications in solid organ transplant recipients, supporting its role in improving post-transplant outcomes. Future studies should define the optimal timing of bariatric surgery relative to transplantation.

## 1. Introduction

There is still notable stigma concerning bariatric surgery in solid organ transplant candidates and recipients [1]. This stigma is rooted in long-standing concerns regarding excessive perioperative risk and doubts about long-term graft safety. These perceptions have historically made both transplant teams and patients hesitant to consider bariatric surgery as part of routine transplant care.

Patients who are candidates for bariatric surgery are typically more prone to a range of comorbidities, including adipose tissue hyperplasia and dysfunction, metabolic dysregulation, and multiple-organ failure [2,3]. This atypical collection of immune mediators, in addition to adipose tissue hypertrophy damaging capillaries and compromising blood supply, has been mechanistically proven to place bariatric surgery candidates at increased risk of hypoxia, impaired wound healing, and graft infection [4]. These patients also tend to have a more complex body anatomy, and therefore challenges with patient positioning and intraoperative imaging significantly increase operative time [5]. These risk factors often contribute to physician hesitation in offering organ transplantation, despite knowing the heightened health risks associated with unmanaged obesity and organ failure [6,7]. As a result, there is a significant mismatch between the need for surgery in bariatric patients and its availability. Studies have found that among patients awaiting kidney transplantation (KT), their transplant probability declines progressively with increasing body mass index (BMI) [8].

Bariatric surgery is a viable solution to improve transplant outcomes. Weight-loss surgery is proven to mitigate the chronic inflammatory state that causes adverse surgical outcomes and the risk of organ rejection in patients with obesity [9]. Recent studies demonstrate that concurrent liver transplantation and sleeve gastrectomy (SG) in patients who might otherwise not be eligible for transplant is successful in preventing recurrent obesity and mitigating graft failure [10]. However, most of these studies have been performed in sample sizes limited to single centers or without a detailed identification of United States (US) solid organ recipients.

Using a large, national cohort with propensity score–matched controls, we therefore sought to assess graft-related outcomes and identify predictors of graft-related complications in solid organ transplant recipients who did or did not undergo subsequent bariatric surgery. By providing contemporary, real-world estimates of risk, our goal is to challenge persistent stigma and more clearly define the role of bariatric surgery in the care of transplant candidates and recipients.

## 2. Materials and Methods

### 2.1. Data Source and Study Population

This retrospective cohort study evaluated transplant-related outcomes in solid organ transplant recipients, comparing those who underwent bariatric surgery with matched controls who did not undergo bariatric surgery. We utilized the Nationwide Inpatient Sample (NIS) database, which is the largest publicly accessible, all-payer database of inpatient hospitalizations in the United States. The analysis encompassed admissions between 2015 and 2020. The NIS is part of the Healthcare Cost and Utilization Project (HCUP) managed by the Agency for Healthcare Research and Quality (AHRQ), and includes data from approximately 20% of US hospitals, encompassing over 7 million hospitalizations each year. Each record is assigned a discharge weight that allows for national estimates, yielding an annual representation of approximately 35 million hospital discharges across the United States.

This database provides detailed demographic, clinical, and hospital-level information, including age, sex, race, household income quartile by ZIP code, hospital region, total charges, and length of stay (LOS). Diagnoses and procedures were identified using the International Classification of Diseases, Tenth Revision, Clinical Modification and Procedure Coding System (ICD-10-CM/PCS).

### 2.2. Study Group Definitions

Two study groups were defined based on bariatric surgery history following solid organ transplantation. The exposure group (solid transplant plus bariatric surgery) included adult patients with a documented solid organ transplant status code (Z94.0, Z94.1, Z94.2, Z94.4, or Z94.83) and a concurrent diagnosis code indicating history of bariatric surgery (Z98.84). The control group (solid transplant only) included adult patients with a documented solid organ transplant status code, but without any history of bariatric surgery. Bariatric surgery history was identified using ICD-10-CM diagnosis code Z98.84, which captures patients with a personal history of weight-loss surgery including Roux-en-Y gastric bypass (RYGB), sleeve gastrectomy (SG), adjustable gastric banding, biliopancreatic diversion (with or without duodenal switch), and one-anastomosis gastric bypass. The NIS database does not provide procedure-specific codes that would allow stratification by individual bariatric surgery type; therefore, our analysis encompasses all bariatric procedures collectively.

### 2.3. Inclusion and Exclusion Criteria

Our study included adult patients (≥18 years) with a diagnosis code indicating solid organ transplantation status (kidney, liver, heart, lung, or pancreas).

Exclusion criteria included hospitalizations with incomplete demographic data (missing age, sex, or income information), records with missing outcome variables, and patients younger than 18 years. To ensure independence of observations, interhospital transfers were also excluded.

**Figure 1:**
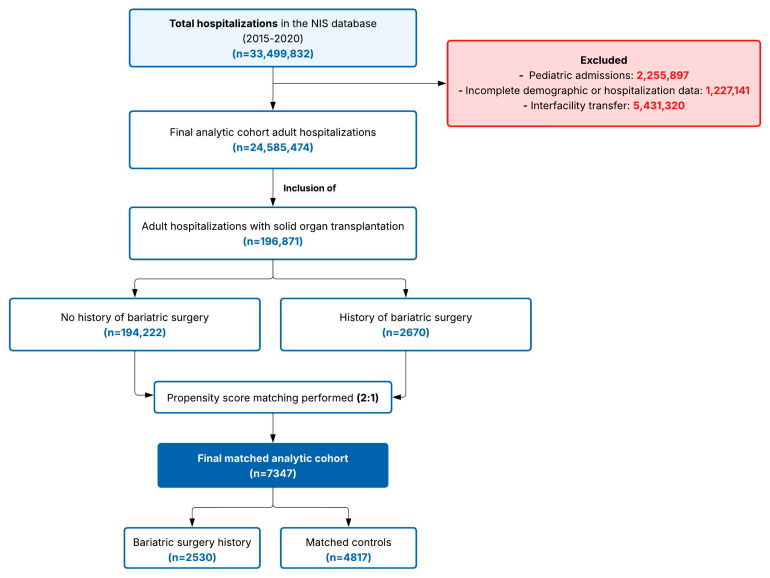
Study flow diagram showing participant selection and propensity score matching process. Selection of study participants from the Nationwide Inpatient Sample (2015–2020). In sum, 196,871 adult solid organ transplant hospitalizations were identified: 2670 had bariatric surgery history and 194,222 did not. After 2:1 propensity score matching on key covariates, the final cohort included 7347 patients: 2530 with bariatric surgery history and 4817 matched controls. Abbreviation: NIS, Nationwide Inpatient Sample.

Notably, we did not exclude patients with obesity at admission who also had a history of bariatric surgery. This decision was made to avoid introducing selection bias, as excluding such patients would assume that all individuals remain obese following bariatric surgery throughout all subsequent hospitalizations, which does not reflect the clinical reality of variable weight trajectories post-bariatric surgery. Obesity status (E66.x), however, was considered in the propensity match, achieving perfect balance between the groups, to reduce the bias the condition might introduce. A detailed flow diagram of hospitalization-type selection is provided in Figure 1.

### 2.4. Covariates

Baseline demographic and clinical characteristics included age, sex, race/ethnicity, income quartile by ZIP code, hospital region, and organ type. Comorbidity burden was assessed using the Charlson Comorbidity Index (CCI). Specific comorbidities analyzed included coronary artery disease, congestive heart failure, peripheral vascular disease, chronic obstructive pulmonary disease, chronic kidney disease, liver disease, diabetes, obesity, hypertension, hyperlipidemia, atrial fibrillation, depression, alcohol use, tobacco use, any malignancy, acute kidney injury, urinary tract infection, sepsis, and opportunistic infection. Hospital-level outcomes included LOS and total hospitalization charges.

### 2.5. Outcomes

Primary outcome: Composite graft-related complications, including acute graft rejection, chronic graft rejection, graft failure, cardiac allograft vasculopathy, or organ-specific transplant complications (kidney, heart, lung, liver, and pancreas).

Secondary outcomes: Individual graft-related complications (acute graft rejection, chronic graft rejection, graft failure, and cardiac allograft vasculopathy), organ-specific transplant complications (kidney, heart, lung, liver, and pancreas), hospitalization burden defined by LOS and total hospitalization charges, and comorbidity prevalence.

### 2.6. Propensity Score Matching

To minimize selection bias and balance baseline characteristics between the groups, 2:1 propensity score matching was performed. Matching was performed using a nearest-neighbor algorithm without replacement and a caliper width of 0.1 of the standard deviation of the logit of the propensity score. Matching was based on key demographic and clinical covariates, including age, sex, race, zip-code median household income, Charlson Comorbidity Index, and transplanted organ type.

### 2.7. Statistical Analysis

Categorical variables are expressed as frequencies and percentages and compared using Pearson’s chi-squared test or Fisher’s exact test when appropriate. Continuous variables are reported as means with standard deviation (SD) and compared using the t test. Standardized mean differences (SMDs) were calculated to assess post-matching balance between groups, with an SMD < 0.1 indicating acceptable balance.

To identify independent predictors of composite graft-related complications, univariate logistic regression was first performed to assess the association between each covariate and the outcome. Variables with clinical relevance or those demonstrating associations on univariate analysis were then included in a multivariate logistic regression model. Covariates included in the multivariate model were bariatric surgery history, age, sex, race/ethnicity, chronic obstructive pulmonary disease, acute kidney injury, and sepsis. Results are reported as odds ratios (ORs) with 95% confidence intervals (CIs).

All analyses were performed using Stata version 19.0 (StataCorp LP, College Station, TX, USA). Statistical significance was defined as a two-tailed *p* value < 0.05.

### 2.8. Ethical Considerations

The NIS is a de-identified, publicly available dataset. Therefore, this study was exempt from institutional review board approval and the requirement for informed consent.

## 3. Results

### 3.1. Study Population and Propensity Score Matching

The initial study population comprised 196,871 patients with a history of solid organ transplantation, of whom 2670 (1.4%) had a documented history of bariatric surgery and 194,232 (98.6%) did not. Following 2:1 propensity score matching, the final analytic cohort comprised 7347 patients: 2530 patients with a history of bariatric surgery and 4817 matched controls without bariatric surgery (Table 1).

### 3.2. Baseline Characteristics of the Matched Cohort

After propensity score matching, the bariatric surgery and control groups demonstrated well-balanced covariate distributions. The mean age at admission was similar between groups: 55.60 (SD 11.30) years in the bariatric surgery group versus 56.26 (SD 13.93) years in controls (*p* = 0.041; SMD = −0.05). Sex distribution was comparable, with females comprising 37.5% of the bariatric surgery group and 36.0% of controls (*p* = 0.20; SMD = −0.03). Racial composition was similar between groups, with White patients representing 63.2% and 67.9%, Black patients 21.9% and 18.4%, and Hispanic patients 11.3% and 10.7% in the bariatric surgery and control groups, respectively. Median household income quartile distribution was well balanced across groups (*p* = 0.504; SMD = 0.04).

Both groups had comparable comorbidity burden, measured by the CCI, with mean scores of 3.27 (SD 1.8) in the bariatric surgery group and 3.28 (SD 1.8) in controls (*p* = 0.94; SMD = 0). The distribution of transplanted organ type was similar after matching, with kidney transplants being most common in both groups (63.6% vs. 60.7%), followed by liver (26.0% vs. 26.0%), heart (9.3% vs. 8.0%), lung (3.1% vs. 5.7%), and pancreas (4.2% vs. 7.9%). Hospital region distribution showed most patients in both groups were from the South (37.4% vs. 38.2%) and Midwest (27.9% vs. 24.8%) regions (Table 1).

### 3.3. Comorbidity Comparison

After propensity score matching, several comorbidities differed significantly between groups (Table 2).

Patients with a history of bariatric surgery had significantly lower rates of coronary artery disease (20.8% vs. 25.3%, *p* < 0.001), congestive heart failure (15.8% vs. 19.2%, *p* < 0.001), chronic obstructive pulmonary disease (6.4% vs. 8.2%, *p* = 0.01), hypertension (75.3% vs. 77.6%, *p* = 0.03), and sepsis (12.5% vs. 14.4%, *p* = 0.02) compared to controls.

Conversely, patients in the bariatric surgery group had significantly higher rates of depression (22.8% vs. 16.6%, *p* < 0.001) and alcohol use (4.7% vs. 2.1%, *p* < 0.001). No significant differences were observed in the prevalence of peripheral vascular disease, chronic kidney disease, liver disease, diabetes, hyperlipidemia, atrial fibrillation, tobacco use, steroid use, any malignancy, acute kidney injury, urinary tract infection, or opportunistic infection between groups.

### 3.4. Hospitalization Outcomes

LOS was similar between the bariatric surgery and control groups, with mean values of 5.46 (SD 6.27) days and 5.30 (SD 5.16) days, respectively (*p* < 0.001; SMD = 0.11). Total hospitalization charges did not differ significantly between groups, with mean charges of USD 75,486 (SD 140,059) in the bariatric surgery group compared to USD 70,722 (SD 142,375) in controls (*p* = 0.17; SMD = 0.03).

### 3.5. Graft-Related Complications

**Figure 2:**
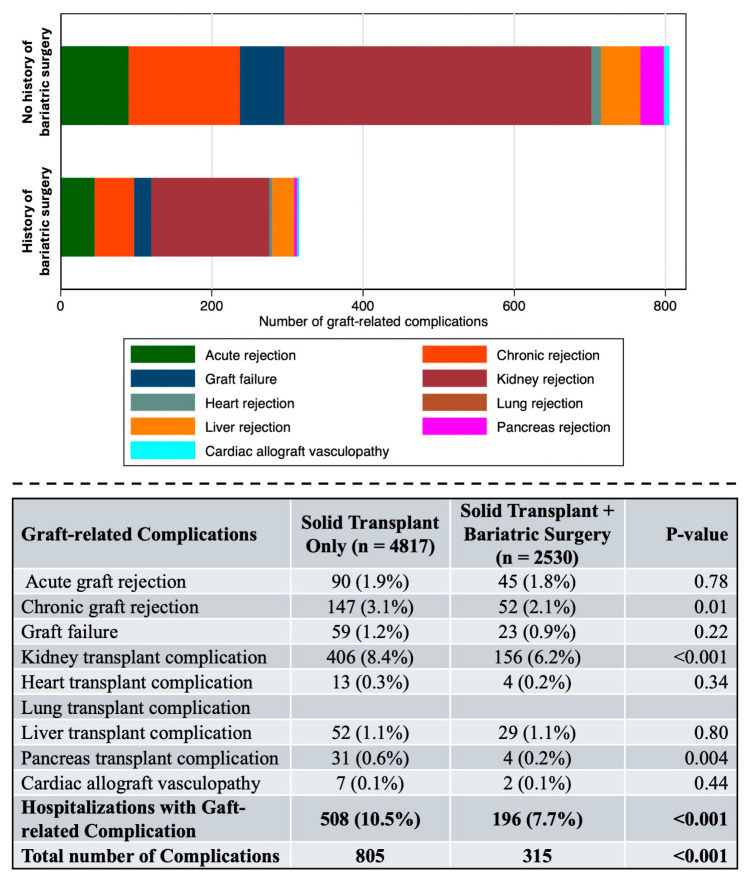
Graft-related complications within the propensity score–matched study cohort. Complications in solid organ transplant recipients by bariatric surgery history. Stacked bar chart (upper panel) and summary (lower panel) displaying graft-related complications in the propensity score–matched cohort. Patients with a history of bariatric surgery (*n* = 2530) had significantly fewer composite graft-related complications compared to controls (*n* = 4817) (7.7% vs. 10.5%, *p* < 0.001). Bold letters indicate the summatory of all the complications. Bariatric surgery was associated with lower rates of chronic graft rejection (2.1% vs. 3.1%, *p* = 0.01), kidney transplant complications (6.2% vs. 8.4%, *p* < 0.001), and pancreas transplant complications (0.2% vs. 0.6%, *p* = 0.004). No significant differences were observed for acute graft rejection, graft failure, heart, lung, or liver transplant complications.

Patients with a history of bariatric surgery experienced significantly fewer composite graft-related complications compared to matched controls (7.7% vs. 10.5%, *p* < 0.001) (Figure 2).

The total number of graft-related complications was also significantly lower in the bariatric surgery group (315 vs. 805 complications, *p* < 0.001).

Analysis of individual graft-related complications revealed that bariatric surgery was associated with significantly lower rates of chronic graft rejection (2.1% vs. 3.1%, *p* = 0.01) and kidney transplant complications (6.2% vs. 8.4%, *p* < 0.001). Pancreas transplant complications were also significantly lower in the bariatric surgery group (0.2% vs. 0.6%, *p* = 0.004). Rates of acute graft rejection (1.8% vs. 1.9%, *p* = 0.78), graft failure (0.9% vs. 1.2%, *p* = 0.22), heart transplant complications (0.2% vs. 0.3%, *p* = 0.34), liver transplant complications (1.1% vs. 1.1%, *p* = 0.80), and cardiac allograft vasculopathy (0.1% vs. 0.1%, *p* = 0.44) were comparable between groups.

### 3.6. Predictors of Graft-Related Complications

**Figure 3:**
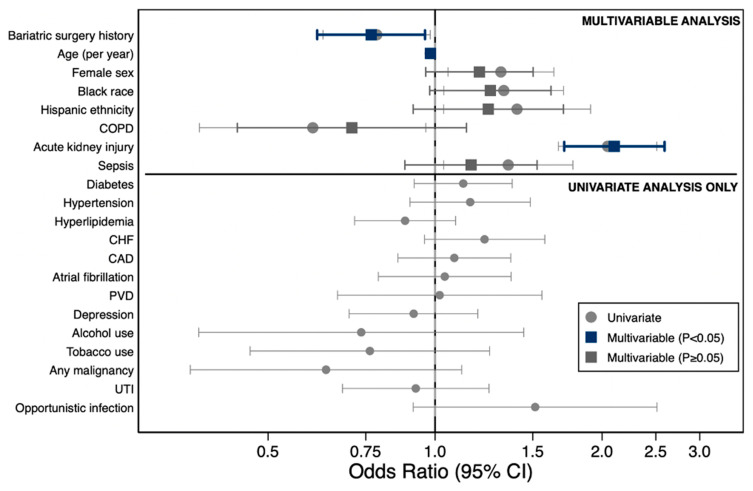
Factors Associated with graft-related complications following liver transplantation. Forest plot displaying odds ratios (ORs) with 95% confidence intervals for factors associated with graft-related complications. Upper panel: multivariate model (circles, univariate; squares, multivariate adjusted; dark blue, *p* < 0.05; gray, *p* ≥ 0.05). Lower panel: univariate analysis only. Acute kidney injury (OR, 2.05) and Black race (OR, 1.35) were independently associated with increased risk, whereas bariatric surgery history (OR, 0.72) and COPD (OR, 0.78) were protective. Abbreviations: CAD, coronary artery disease; CHF, congestive heart failure; CI, confidence interval; COPD, chronic obstructive pulmonary disease; PVD, peripheral vascular disease; UTI, urinary tract infection.

On univariate logistic regression analysis, several factors were significantly associated with graft-related complications (Table 3, Figure 3).

Age was inversely associated with complications (OR 0.98, 95% CI 0.97–0.99, *p* < 0.001), while female sex (OR 1.31, 95% CI 1.05–1.64, *p* = 0.015), Black race (OR 1.33, 95% CI 1.04–1.70, *p* = 0.024), Hispanic ethnicity (OR 1.41, 95% CI 1.04–1.91, *p* = 0.029), acute kidney injury (OR 2.05, 95% CI 1.67–2.51, *p* < 0.001), and sepsis (OR 1.35, 95% CI 1.04–1.77, *p* = 0.027) were positively associated with increased risk. Chronic obstructive pulmonary disease (COPD) was associated with reduced risk on univariate analysis (OR 0.60, 95% CI 0.38–0.96, *p* = 0.034).

On multivariate logistic regression analysis adjusting for age, sex, race/ethnicity, COPD, acute kidney injury, and sepsis, bariatric surgery history was independently associated with a 23% reduction in the odds of graft-related complications (OR 0.77, 95% CI 0.61–0.96, *p* = 0.020). Age remained inversely associated with complications (OR 0.98, 95% CI 0.97–0.99, *p* < 0.001), and acute kidney injury remained the strongest predictor of graft-related complications (OR 2.10, 95% CI 1.71–2.59, *p* < 0.001). Female sex, Black race, Hispanic ethnicity, COPD, and sepsis were no longer statistically significant in the adjusted model (Table 3, Figure 2).

## 4. Discussion

In this large, propensity-matched cross-sectional study, our findings indicate that patients with a history of bariatric surgery demonstrated significantly lower rates of metabolic-associated comorbidities, including coronary artery disease, congestive heart failure, and hypertension. Notably, bariatric surgery was independently associated with 23% reduced odds of organ graft complications after adjusting for multiple cardiometabolic risk factors. Despite these findings, bariatric surgery continues to be an underutilized resource in patients in need of life-saving organ transplantation.

Bariatric surgery has been identified as the most cost-effective strategy for kidney transplant candidates with obesity at a willingness-to-pay (WTP) threshold of USD 50,000 per quality-adjusted life year [11]. This cost reduction can be attributed to a lower incidence of post-transplant complications, resulting in reduced use of intensive care services and medications. In contrast, another well-designed study, although with limited sample size, found no significant difference in hospitalization costs among a cohort of 39 liver transplant recipients who had previously undergone bariatric surgery compared to controls. Our study similarly found comparable expenses between patients undergoing and not undergoing bariatric surgery. The variability of the current data on this topic might be explained due to the variable sample size of the studies. However, the population-level sample size in this study strengthens the validity of the findings [12].

Allograft rejection is mediated by both innate and adaptive immune activation, a process that is exacerbated in the context of obesity [13]. Bariatric surgery may therefore attenuate this heightened immune response and reduce the risk of allograft rejection. In the present study, we observed that bariatric surgery was associated with a significant reduction in overall graft complications among solid organ transplant recipients, including chronic graft rejection and organ-specific complications such as in kidney and pancreas grafts. These findings are consistent with those of prior reports that bariatric surgery performed after KT was associated with a 15% reduced risk of all-cause graft failure, predominantly driven by decreases in chronic rejection [14]. In contrast, studies have found no significant differences in graft loss between KT recipients with obesity who underwent laparoscopic SG and BMI-matched controls, underscoring a possible procedure-specific association, though this was limited by small samples [15,16].

Evidence indicates heterogeneity in outcomes by bariatric procedure type. A US study reported that RYGB was associated with higher odds of overall morbidity, surgery-related complications, and hospital readmissions compared to SG among transplant recipients [17]. These differences likely reflect procedure-specific effects. RYGB involves intestinal bypass with significant implications for drug and nutrient absorption, whereas SG preserves intestinal continuity [18,19]. Consequently, emerging evidence suggests that SG has become the preferred bariatric approach in transplant populations, owing to more reliable immunosuppressant absorption and a lower risk of micronutrient deficiencies [18,19].

Nonetheless, like all bariatric procedures, SG carries a risk of micronutrient deficiencies, which typically emerge within 10 years postoperatively [20]. These deficiencies are particularly consequential in transplant recipients given the increased metabolic demands of graft function [21,22]. Notably, vitamin D deficiency has been associated with high odds of acute rejection in KT recipients (OR 1.82; 95% CI 1.29–2.56) [23]. However, evidence indicates that structured supplementation and routine monitoring of hematologic indices, micronutrient levels, and bone density can effectively mitigate these deficiencies [24,25].

Concerns have been raised regarding the potential impact of bariatric surgery on immunosuppressant absorption and subsequent graft function [26,27,28]. A study evaluating the pharmacokinetics of tacrolimus, sirolimus, and mycophenolic acid in a cohort of KT recipients and candidates who had undergone RYGB [29] demonstrated reduced area under the concentration–time curve (AUC) for all three drugs, suggesting that higher doses may be required to achieve adequate immunosuppression in this population. In contrast, a US prospective study reported stable immunosuppressive drug levels in transplant recipients who underwent laparoscopic SG or RYGB, with no need for significant dose adjustments and no incidents of graft loss or major complications [19]. Similarly, more evidence has shown no significant changes in tacrolimus blood levels or dosing requirements in transplant recipients following laparoscopic SG or RYGB, indicating that both procedures can maintain effective immunosuppression post-transplant [30]. Our cross-sectional study favors that immunosuppression for transplant recipients is unaffected by bariatric surgery, though further studies supporting its efficacy are still needed.

The timing of bariatric surgery relative to transplantation is a clinically important consideration, as it may influence patient and graft outcomes. Although the benefits of bariatric surgery in solid organ transplantation are well established, the question of the most advantageous timing remains unresolved. A systematic review encompassing 19 studies evaluating patients who underwent bariatric surgery before or after liver transplant reported no 30-day mortality in either group, reinforcing the overall safety and potential benefit of the procedure regardless of timing [31]. Similarly, reductions in all-cause allograft failure and mortality have been seen in KT recipients who underwent bariatric surgery regardless of timing (before or after surgery) [14]. Although we recognize this is a critical question to answer, the cross-sectional nature of the NIS database precluded assessment of the optimal longitudinal assessment of bariatric surgery relative to transplantation, and larger, well-designed prospective trials are needed to more definitively address this question.

The most actionable interpretation of our study is that a history of bariatric surgery is independently associated with reduced odds of graft-related complications after adjustment for multiple baseline risk factors. These findings align with the growing body of evidence supporting the safety and potential benefits of bariatric surgery in the transplant population [14]. Regarding comorbidities, we observed significantly lower rates of coronary artery disease, congestive heart failure, and hypertension among transplant recipients with a history of bariatric surgery. Studies have shown similar results in a cohort of seven heart transplant candidates, demonstrating higher benefits in left ventricular ejection fraction (LVEF) among those who underwent SG [32]. These findings further underscore the cardiovascular benefits of bariatric surgery in the transplant population, which may contribute to the observed reduction in graft-related complications through attenuation of the chronic inflammatory state and improved metabolic profiles associated with sustained weight loss.

This study has several limitations. First, the NIS only captures inpatient data; therefore, long-term post-discharge outcomes, graft survival beyond hospitalization, readmission rates, and mortality were not available. The database provides an unknown time elapsed between transplant and bariatric surgery, but prior studies have demonstrated comparable safety and efficacy regardless of whether bariatric surgery occurs before or after transplantation. Second, although the database captures multiple bariatric procedures (e.g., RYGB, SG, gastric banding), insufficient procedural granularity precluded stratification of outcomes by specific surgery type. Given emerging evidence that SG and RYGB differ in immunologic impact, nutritional effects, and drug absorption, future procedure-specific analyses are warranted. Third, the NIS does not include information on immunosuppressive regimens, drug levels, adherence, or adjustments following bariatric surgery, limiting interpretation of how these factors may influence graft outcomes. Specifically, we could not assess corticosteroid use, calcineurin inhibitor regimens, or other maintenance immunosuppression protocols that may impact outcomes. Fourth, the NIS database does not include ASA physical status classification scores or other procedure-specific risk stratification tools that allow a more detailed perioperative risk assessment. However, we used the Charlson Comorbidity Index as a validated proxy for overall comorbidity burden, which was well balanced between groups after propensity score matching.

## 5. Conclusions

In this nationally representative cohort of propensity score–matched solid organ transplant recipients, bariatric surgery was associated with significantly lower rates of composite graft-related complications, driven primarily by reductions in chronic graft rejection, kidney transplant complications, and pancreas transplant complications. Additionally, patients with prior bariatric surgery demonstrated lower prevalence of cardiovascular comorbidities, including coronary artery disease, congestive heart failure, and hypertension. These findings reinforce the role of bariatric surgery as a safe and potentially immunomodulatory strategy to improve outcomes in a population historically excluded from transplantation due to perceived surgical risk.

Future work should focus on prospective studies that can corroborate our findings and evaluate long-term survival, graft function, and longitudinal quality of life outcomes, stratifying by specific bariatric procedure type. The integration of bariatric surgery and obesity specialists into the multidisciplinary organ transplantation team can help mitigate provider bias and optimize perioperative care of patient candidates for both bariatric surgery and organ transplantation.

## Figures and Tables

**Table 1:** Baseline and clinical characteristics of the pre- and post-propensity score–matched study cohort.

	Before Propensity Score Matching				After Propensity Score Matching			
Characteristic	Solid Transplant Only	Solid Transplant + Bariatric	*p*	SMD	Solid Transplant Only	Solid Transplant + Bariatric	*p*	SMD
*n*	194,232 (98.6)	2670 (1.4)			4817 (65.6)	2530 (34.4)		
Age, years, mean (SD)	57.7 (15.4)	55.6 (11.3)	<0.001	−0.15	56.3 (13.9)	55.6 (11.3)	0.041	−0.05
Length of stay, days, mean (SD)	6.2 (9.3)	5.5 (6.3)	<0.001	−0.09	5.3 (5.2)	5.5 (6.3)	<0.001	0.11
Total hospitalization charge, USD, mean (SD)	90,716 (204,672)	75,464 (139,219)	<0.001	−0.09	70,722 (142,375)	75,486 (140,059)	0.17	0.03
Sex			<0.001	0.43			0.20	−0.03
Female	113,606 (58.5)	997 (37.3)			1732 (36.0)	948 (37.5)		
Male	80,588 (41.5)	1673 (62.7)			3085 (64.0)	1582 (62.5)		
Race/ethnicity			<0.001	−0.13			<0.001	0.09
White	113,178 (60.3)	1618 (63.0)			3271 (67.9)	1600 (63.2)		
Black	35,260 (18.8)	560 (21.8)			885 (18.4)	554 (21.9)		
Hispanic	25,704 (13.7)	293 (11.4)			517 (10.7)	285 (11.3)		
Asian	6582 (3.5)	26 (1.0)			79 (1.6)	24 (0.9)		
Native American	1488 (0.8)	12 (0.5)			15 (0.3)	10 (0.4)		
Other	5557 (3.0)	58 (2.3)			50 (1.0)	57 (2.3)		
Median household income quartile, USD			<0.001	0.09			0.504	0.04
1–24,999	52,451 (27.4)	595 (22.6)			1175 (24.4)	580 (22.9)		
25,000–34,999	49,010 (25.6)	709 (26.9)			1295 (26.9)	678 (26.8)		
35,000–44,999	48,452 (25.3)	713 (27.1)			1264 (26.2)	681 (26.9)		
45,000 or more	41,369 (21.6)	614 (23.3)			1083 (22.5)	591 (23.4)		
Charlson Comorbidity Index, mean (SD)	3.6 (2.0)	3.3 (1.9)	<0.001	−0.15	3.3 (1.8)	3.3 (1.8)	0.94	<0.001
Hospital region			<0.001	−0.03			<0.001	0.05
Northeast	37,361 (19.2)	437 (16.4)			970 (20.1)	427 (16.9)		
Midwest	45,135 (23.2)	783 (29.3)			1196 (24.8)	705 (27.9)		
South	73,398 (37.8)	981 (36.7)			1839 (38.2)	945 (37.4)		
West	38,338 (19.7)	469 (17.6)			812 (16.9)	453 (17.9)		
Organ type								
Kidney	115,889 (59.7)	1687 (63.2)	<0.001	0.07	2924 (60.7)	1609 (63.6)	0.16	0.06
Heart	16,929 (8.7)	249 (9.3)	0.27	0.02	383 (8.0)	236 (9.3)	0.40	0.05
Lung	10,851 (5.6)	83 (3.1)	<0.001	−0.12	274 (5.7)	78 (3.1)	0.06	−0.13
Liver	51,794 (26.7)	699 (26.2)	0.58	−0.01	1254 (26.0)	657 (26.0)	0.95	0
Pancreas	12,900 (6.6)	110 (4.1)	<0.001	−0.11	381 (7.9)	105 (4.2)	<0.001	−0.16

Values are presented as *n* (%) or mean (SD). Abbreviations: SD, standard deviation; SMD, standardized mean difference; USD, United States dollars.

**Table 2:** Comparison of comorbidities within the propensity score–matched study cohort.

Comorbidity	No Bariatric Surgery (*n* = 4817)	Bariatric Surgery (*n* = 2530)	*p* Value	SMD
Coronary artery disease	1217 (25.3)	527 (20.8)	<0.001	−0.11
Congestive heart failure	924 (19.2)	400 (15.8)	<0.001	−0.09
Peripheral vascular disease	305 (6.3)	135 (5.3)	0.09	−0.04
Chronic obstructive pulmonary disease	397 (8.2)	162 (6.4)	0.01	−0.07
Chronic kidney disease	2651 (55.0)	1335 (52.8)	0.06	−0.05
Liver disease	166 (3.4)	94 (3.7)	0.55	0.01
Diabetes mellitus	2339 (48.6)	1262 (49.9)	0.28	0.03
Obesity	1402 (29.1)	733 (29.0)	0.10	0.01
Hypertension	3738 (77.6)	1906 (75.3)	0.03	−0.05
Hyperlipidemia	1959 (40.7)	987 (39.0)	0.17	−0.03
Atrial fibrillation	765 (15.9)	391 (15.5)	0.63	−0.01
Depression	801 (16.6)	578 (22.8)	<0.001	0.16
Alcohol use disorder	103 (2.1)	120 (4.7)	<0.001	0.14
Tobacco use	275 (5.7)	130 (5.1)	0.31	−0.03
Steroid use	733 (15.2)	443 (17.5)	0.07	0.1
Any malignancy	253 (5.3)	114 (4.5)	0.16	−0.03
Acute kidney injury	1543 (32.0)	822 (32.5)	0.69	0.01
Urinary tract infection	653 (13.6)	343 (13.6)	1.00	<0.001
Sepsis	695 (14.4)	316 (12.5)	0.02	−0.06
Opportunistic infection	131 (2.7)	87 (3.4)	0.08	0.04

Values are presented as *n* (%). Abbreviation: SMD, standardized mean difference.

**Table 3:** Univariate and multivariate logistic regression analysis of factors associated with graft complications.

	Univariate Analysis		Multivariate Analysis	
Variable	OR (95% CI)	*p* Value	OR (95% CI)	*p* Value
Bariatric surgery history	0.78 (0.62–0.98)	0.01	0.77 (0.61–0.96)	0.02
Age (per year)	0.98 (0.97–0.99)	<0.001	0.98 (0.97–0.99)	<0.001
Female sex	1.31 (1.05–1.64)	0.015	1.20 (0.96–1.50)	0.11
Race/ethnicity				
White	Reference		Reference	
Black	1.33 (1.04–1.70)	0.02	1.26 (0.98–1.62)	0.074
Hispanic	1.41 (1.04–1.91)	0.03	1.25 (0.91–1.70)	0.165
Asian	1.20 (0.52–2.77)	0.66	—	—
Other	0.76 (0.28–2.07)	0.59	—	—
Diabetes mellitus	1.12 (0.92–1.38)	0.26	—	—
Hypertension	1.16 (0.90–1.48)	0.25	—	—
Hyperlipidemia	0.88 (0.72–1.09)	0.25	—	—
Congestive heart failure	1.23 (0.96–1.58)	0.11	—	—
Coronary artery disease	1.08 (0.86–1.37)	0.51	—	—
COPD	0.60 (0.38–0.96)	0.03	0.71 (0.44–1.14)	0.154
Atrial fibrillation	1.04 (0.79–1.37)	0.78	—	—
Peripheral vascular disease	1.02 (0.67–1.56)	0.93	—	—
Depression	0.91 (0.70–1.19)	0.51	—	—
Alcohol use disorder	0.74 (0.37–1.45)	0.37	—	—
Tobacco use	0.76 (0.46–1.25)	0.29	—	—
Acute kidney injury	2.05 (1.67–2.51)	<0.001	2.10 (1.71–2.59)	<0.001
Any malignancy	0.64 (0.36–1.12)	0.12	—	—
Urinary tract infection	0.92 (0.68–1.25)	0.61	—	—
Sepsis	1.35 (1.04–1.77)	0.03	1.16 (0.88–1.53)	0.287
Opportunistic infection	1.51 (0.91–2.51)	0.11	—	—

Abbreviations: CI, confidence interval; COPD, chronic obstructive pulmonary disease; OR, odds ratio. Note: Multivariate model included variables with *p* < 0.10 on univariate analysis. Em dash (—) indicates variable not included in the multivariate model.

## Data Availability

This study utilized the Nationwide Inpatient Sample (NIS), a publicly available, de-identified database maintained by the Healthcare Cost and Utilization Project (HCUP). Data can be accessed through the HCUP after completion of the required data use agreement and purchase of the dataset (https://www.hcup-us.ahrq.gov (accessed on 5 July 2025)).

## Abbreviations

The following abbreviations are used in this manuscript.

AHRQ	Agency for Healthcare Research and Quality
AUC	area under the concentration–time curve
BMI	body mass index
CCI	Charlson Comorbidity Index
CI	confidence interval
CM	clinical modification
COPD	chronic obstructive pulmonary disease
HCUP	Healthcare Cost and Utilization Project
ICD-10-CM	International Classification of Diseases, Tenth Revision, Clinical Modification
ICD-10-PCS	International Classification of Diseases, Tenth Revision, Procedure Coding System
KT	kidney transplantation
LOS	length of stay
LVEF	left ventricular ejection fraction
NIS	Nationwide Inpatient Sample
OR	odds ratio
PCS	Procedure Coding System
RYGB	Roux-en-Y gastric bypass
SD	standard deviation
SG	sleeve gastrectomy
SMD	standardized mean difference
US	United States
WTP	willingness to pay
